# Multiple Parallel Hits Hypothesis in Nonalcoholic Fatty Liver Disease: Revisited After a Decade

**DOI:** 10.1002/hep.31518

**Published:** 2021-02-06

**Authors:** Herbert Tilg, Timon E. Adolph, Alexander R. Moschen

**Affiliations:** ^1^ Department of Internal Medicine I Gastroenterology Hepatology Endocrinology & Metabolism Medical University Innsbruck Innsbruck Austria

**Abbreviations**
AT: adipose tissue
ATM: AT macrophage
CCL: C‐C motif chemokine ligand
CD: cluster of differentiation
IL: interleukin
KO: knockout
NAFLD: nonalcoholic fatty liver disease
NASH: nonalcoholic steatohepatitis
NK: natural killer
SAT: subcutaneous AT
T2D: type 2 diabetes
TMAO: trimethylamine N‐oxide
TNFα: tumor necrosis factor alpha
VAT: visceral AT

Nonalcoholic fatty liver disease (NAFLD) is an epidemic liver disease, affecting approximately one quarter of the entire population in the world.^(^ ^1^ ^)^ This disease encompasses a broad spectrum of clinical phenotypes ranging from hepatic steatosis to nonalcoholic steatohepatitis (NASH), fibrotic NASH, advanced fibrosis, liver cirrhosis, and hepatocellular carcinoma (HCC). Although liver inflammation in NAFLD appears less prognostically relevant when compared to liver fibrosis,^(^ ^2^ ^)^ the latter may be the cumulative result of the former.^(^ ^3^ ^)^ Noninvasive assessment of hepatic fibrosis (e.g., by transient elastography) has reduced the need for invasive procedures such as liver biopsy,^(^ ^4^ ^)^ although late‐stage clinical trials still require histologic endpoints.

The liver plays a crucial role in glucose and lipid metabolism. NAFLD is frequently present in obesity and reflects a risk factor for many metabolic diseases such as type 2 diabetes (T2D).^(^ ^5^ ^)^ In turn, T2D is associated with NAFLD in up to 90% of patients. NAFLD has been linked to various extrahepatic disorders such as cardiovascular complications^(^ ^6^ ^)^ and chronic kidney disease.^(^ ^7^ ^)^ Furthermore, NAFLD is not only a major risk factor for HCC but also associated with an increased rate of various extrahepatic malignancies such as gastrointestinal and gynecological malignancies.^(^ ^4^ ^)^ This cancer risk seems to be even higher in NAFLD than in obesity itself.^(^ ^8^ ^)^ As such, NAFLD reflects a prototypic systemic metabolic disorder targeting and affecting many extrahepatic organs throughout the body.

The pathophysiology underlying this disorder is complex and incompletely understood. A decade ago, we proposed a multiple parallel hits hypothesis in which lipotoxicity of adipose tissue (AT) and alterations in gut microbial functions contribute to the evolution of inflammation and fibrosis in NAFLD.^(^ ^9^ ^)^ Progress over the last decade was substantial in that the role of AT inflammation^(^ ^10^ ^)^ and the gut microbiome (and related metabolites) evolved as crucial players in the pathogenesis of NAFLD.^(^ ^11^, ^12^ ^)^ Furthermore, various dietary components as other gastrointestinal hits with proinflammatory potential have been identified. Finally, genetic pathways also play a role in disease manifestation; and several genetic hits, such as patatin‐like phospholipase domain containing 3, transmembrane 6 superfamily member 2, glucokinase regulator, membrane‐bound O‐acyltransferase domain containing 7, and hydroxysteroid 17‐beta dehydrogenase 13, are involved especially in lipid metabolism.^(^ ^13^ ^)^ In this review, we will discuss pathophysiological factors focusing on the intricate triangular interplay between the gastrointestinal tract, AT, and the liver.

## AT Inflammation as a Driver of NAFLD

### AT Inflammation: From Mediators to Immune Cell Profiles

Normal AT is composed of adipocytes, fibroblasts, endothelial cells, and even resident macrophages and other cells of the immune system which collectively regulate host metabolism and energy storage.^(^ ^14^ ^)^ White AT depots comprise visceral AT (VAT) and subcutaneous AT (SAT), which, together with the liver, participate in fatty acid metabolism. In health, AT communicates with the liver to control energy homeostasis.^(^ ^15^ ^)^ In obesity, AT inflammation is characterized by increased cytokine and chemokine expression and infiltration of immune cells, for example, leukocytes, which may serve as fuel for local and systemic inflammation. As such, the inflammatory state in AT contributes to systemic inflammation, which may deteriorate liver disease and insulin resistance, exemplifying aspects of an AT–liver axis.^(^ ^9^, ^10^ ^)^

**Fig. 1:**
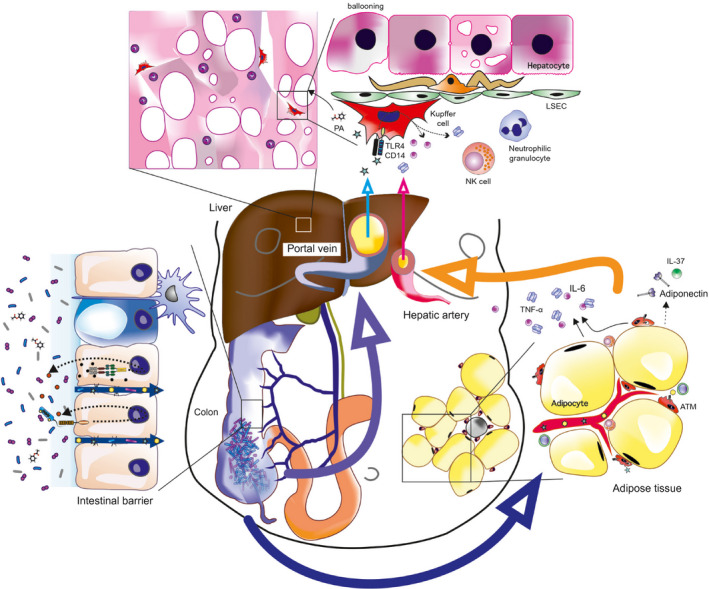
NAFLD and NASH have been associated with alterations in the structural composition of the gut microbiota, with a typical increase in Proteobacteria. Specific microbial signatures have further been related to both hepatic steatosis and fibrosis. Dietary factors and dysbiosis spark subclinical low‐grade intestinal inflammation, resulting in an impaired gut barrier function that facilitates the translocation of bacteria, bacterial components such as lipopolysacchides, microbial metabolites, and gut‐derived immune cells to the liver through the portal circulation. Some bacteria‐derived metabolic products such as phenylacetate trigger hepatic steatosis. The metabolically stressed, insulin‐resistant AT attracts various immune cells including ATMs, T cells, and NK cells. Stimulated by local and gut‐derived factors, there is an increased expression of proinflammatory cytokines including IL‐1β, TNF‐α, and IL‐6, while anti‐inflammatory adipose‐derived factors such as adiponectin and IL‐37 are decreased. The liver represents an important target organ for AT‐derived inflammatory cytokines. The liver has to integrate gut‐derived and AT‐derived signals. Hepatic insulin resistance, increased *de novo* synthesis, and increased dietary and peripheral fatty acid supply promote liver steatosis. Surpassing an individual threshold switches on a vicious cycle characterized by hepatocyte cell death (ballooning), activation of Kupffer and liver sinusoidal endothelial cells (attracting more immune cells), and activation of hepatic stellate cells (promoting liver fibrosis). Furthermore, a gut–AT axis emerges in which microbes, their metabolites, and inflammatory mediators act as fuel for metabolic disease. Abbreviations: LSEC, liver sinusoidal endothelial cell; PA, phenylacetate; TLR4, toll‐like receptor 4.

The hallmarks of AT inflammation are influx of macrophages, cluster of differentiation 4–positive (CD4^+^) and CD8^+^ T cells, dendritic cells, and natural killer (NK) cells and increased expression of cytokines/chemokines.^(^ ^16^ ^)^ Primary cues in AT inflammation remain poorly explored but arguably involve diet‐induced stress of adipocytes, which subsequently induces a cytokine and chemokine response and immune cell infiltration. The initial inflammatory state may be fueled and self‐maintained by tissue‐infiltrating immune cells. For example, recruitment of AT macrophages (ATMs) is dependent on expression of various chemokines such as C‐C motif chemokine ligand 2 (CCL2), expressed by AT in obese animals and patients.^(^ ^17^ ^)^ Adaptive immunity (e.g., T cells) is also recruited to AT by antigen‐presenting functions of adipocytes, which precedes ATM accumulation.^(^ ^18^ ^)^ Expression of various chemokines (besides CCL2) such as CCL5 (also known as regulated upon activation, normal T cell expressed, and secreted) or CCL13 is increased in AT of obese patients.^(^ ^19^ ^)^ Importantly, expression of most of these chemokines is regulated by cytokines such as tumor necrosis factor alpha (TNFα), interleukin 1‐beta (IL‐1β), or IL‐6. TNFα was the first described adipokine associated with obesity‐related insulin resistance in murine models, and its expression is increased in human obesity.^(^ ^20^ ^)^ Similarly, preclinical and clinical evidence indicated a key role for IL‐1β in obesity‐related AT inflammation. IL‐1α‐deficient, IL‐1β‐deficient, and type 1 IL‐1 receptor–deficient mice are protected against high‐fat diet–induced AT inflammation, fatty liver, and insulin resistance^(^ ^21^ ^)^; and human obesity is characterized by increased AT IL‐1β expression.^(^ ^22^ ^)^ IL‐37, an anti‐inflammatory IL‐1 family cytokine member, is highly expressed in AT of obese subjects and is able to improve insulin resistance in experimental models.^(^ ^23^, ^24^ ^)^ IL‐6 is produced in AT, mostly by ATMs and adipocytes.^(^ ^25^ ^)^ The importance of SAT as a source of circulating IL‐6 has been convincingly demonstrated, with 15%‐35% of circulating IL‐6 being derived from this tissue.^(^ ^26^ ^)^ Both SAT and VAT produce large amounts of IL‐6 in obesity and related disorders, and both sources are biologically relevant and affect insulin sensitivity.^(^ ^27^ ^)^ We have investigated AT inflammation in morbidly obese patients undergoing bariatric surgery. Both SAT and VAT highly expressed TNFα, IL‐1β, and IL‐6, which was strongly reduced after successful weight loss.^(^ ^23^, ^28^ ^)^ Similarly, adiponectin and leptin (prototypic immunomodulatory adipokines) are critically involved in AT inflammation and obesity‐related disorders.^(^ ^29^ ^)^ Collectively, these studies highlight the importance of cellular, cytokine, adipokine. and chemokine networks in AT inflammation of obesity‐related disorders (see Fig. 1).

### Clinical Evidence for an AT–Liver Axis

Clinical studies provide evidence that cellular and molecular AT inflammation correlate with degree of liver disease. Du Plessis and colleagues studied transcriptomic profiles in SAT and VAT, functional characteristics of ATMs, and histologic severity of NAFLD in 113 patients undergoing bariatric surgery.^(^ ^30^ ^)^ They found increased expression of inflammatory genes in AT comparing NAFLD and NASH. Furthermore, patients with NASH exhibited an increased number of CD11c^+^CD206^+^ and chemokine (C‐C motif) receptor–positive macrophages in VAT accompanied by increased release of proinflammatory cytokines and chemokines. Most importantly, AT inflammation directly correlated with the degree of liver inflammation.^(^ ^30^ ^)^ Similarly, a large study investigating 3,197 participants observed that NAFLD was independently associated with both subcutaneous and visceral obesity.^(^ ^31^ ^)^ Although these clinical studies provide no proof of concept in human NAFLD, they clearly link AT and liver inflammation, which appears relevant in light of experimental (mechanistic) studies that report evidence for an AT–liver axis in obesity‐related disorders.

Insulin resistance, a hallmark of NAFLD,^(^ ^32^ ^)^ occurs in various tissues such as liver, muscle, and AT.^(^ ^33^ ^)^ While it is commonly conceived that insulin resistance emerges consequent to obesity and systemic inflammation, a recent study demonstrated that insulin resistance may fuel AT inflammation through monocyte chemoattractant protein 1–regulated leukocyte recruitment.^(^ ^34^ ^)^ These findings are interesting as AT inflammation accelerates lipolysis by affecting mitogen‐activated protein kinase (MAPK) signaling, which results in activation of the ß3‐adrenergic receptor.^(^ ^35^ ^)^ Lipolysis will result in an enhanced free fatty acid export to the liver, promoting hepatic steatosis and potentially NAFLD. In line with this, AT insulin resistance correlates with the degree of liver disease (especially fibrosis); and improvement of AT insulin resistance by pioglitazone, a peroxisome proliferator–activated receptor‐gamma agonist, resulted in a decrease in hepatocyte ballooning and liver fibrosis in patients.^(^ ^36^ ^)^ Indeed, AT insulin resistance seems to be linked to the degree of liver inflammation in patients with NAFLD.^(^ ^37^ ^)^ In this study, the authors established a link between AT insulin resistance and liver macrophage activation by measuring soluble CD183, proposing that free fatty acids might be involved. These studies support the concept of crosstalk between insulin resistance and inflammation, especially in AT, which contributes to liver disease.

### Animal Studies Establish an AT–Liver Axis

While the aforementioned clinical studies are notable, they are descriptive and can only provide indirect evidence for an AT–liver axis in mammals. Bijnen and colleagues transplanted VAT from lean, obese, or ATM‐depleted obese mice to lean *Ldr^−/−^* mice.^(^ ^38^ ^)^ AT transplantation from obese mice resulted in increased liver injury and hepatic inflammation, which was less pronounced in ATM‐depleted transplanted AT. Liver injury was paralleled by increased numbers of circulating and hepatic neutrophils, an effect mainly attributed to increased synthesis of the neutrophil chemotaxis proteins chemokine (C‐X‐C motif) ligands 14 and 16 by ATM.^(^ ^38^ ^)^

We previously hypothesized that tissue‐specific knockout (KO) models (e.g., AT‐specific KO mice) would reveal a key role for AT inflammation in NAFLD.^(^ ^9^ ^)^ Indeed, numerous studies have been reported in the last decade that took advantage of adipocyte‐specific KO mouse models; only a few can be discussed here. AT‐specific deletion of insulin receptor and/or insulin‐like growth factor 1 receptor results in severe lipodystrophy accompanied by progressive liver disease resembling NASH, with inflammation, fibrosis, and highly dysplastic liver nodules at week 52.^(^ ^39^ ^)^ Lipid peroxidation in the liver may be a critical mechanism in this model.^(^ ^39^ ^)^ Similarly, AT deficiency of hormone‐sensitive lipase causes liver disease paralleled by an increase in ATMs, lipodystrophy, impaired adipokine synthesis, and evidence for systemic insulin resistance.^(^ ^40^ ^)^ In contrast, AT‐specific deletion of the lipoprotein lipase angiopoietin‐like 4 (which controls fatty acid metabolism) attenuates systemic inflammation, hepatic steatosis, and atherosclerosis.^(^ ^41^ ^)^ We have shown that AT‐specific deletion of type I interferon receptor worsens high‐fat diet–induced metabolic perturbation, indicated by increased weight gain, systemic insulin resistance, and impaired glucose intolerance.^(^ ^42^ ^)^ However, AT‐specific deletion of the type I interferon receptor did not impact the severity of NAFLD in our model. Collectively, these studies indicate that specific cellular hubs control the AT–liver crosstalk in NAFLD that deserve to be dissected in more detail.

## Gastrointestinal Cues Fueling NAFLD: From Intestinal Microbiota to Dietary Factors

Various gastrointestinal factors impinge on the gut–liver axis. The gut–liver crosstalk is shaped not only by the gut microbiome and its metabolites but also by gut hormones and the immune system.^(^ ^43^ ^)^ In this section we discuss the role of the gut microbiome and dietary factors in NAFLD.

### Gut Microbiota in NAFLD: Rheostat of Intestinal Immunity and Hepatic Inflammation

#### Microbiome in NAFLD

Progress has been made over the last decade in deciphering a role for the intestinal microbiota in NAFLD, for example, by identification of a NASH‐associated gut microbiome signature,^(^ ^44^ ^)^ which was preceded by smaller studies that reported gut microbial alterations in patients with NAFLD.^(^ ^45^ ^)^ Collectively, the abundance of Proteobacteria, Enterobacteriaceae, and *Escherichia coli* differed between obese and NASH microbiomes^(^ ^45^ ^)^; and an association was observed with reduced abundance of Bacteroidetes in patients with NASH compared to patients with simple steatosis or healthy controls.^(^ ^46^ ^)^ Boursier and colleagues studied the gut microbiota and severity of histology‐proven NAFLD in 57 patients.^(^ ^47^ ^)^ In this study, *Bacteroides* abundance increased depending on the severity of disease, while *Prevotella* abundance decreased. The most convincing example for a gut microbiome signature in fibrotic NASH comes from a study by Loomba and colleagues.^(^ ^44^ ^)^ These authors investigated 86 histologically defined NAFLD subjects and identified 37 bacterial species, which allowed them to distinguish mild versus advanced fibrosis. Advanced fibrosis was associated with abundance of Proteobacteria and *Escherichia coli* and a decrease in Firmicutes such as *Faecalibacterium prausnitzii*. Such a microbiome signature might be even more prevalent in the case of NASH cirrhosis.^(^ ^48^ ^)^ An important role for bacteria‐derived endotoxin as a disease‐contributing factor had been claimed already more than 20 years ago.^(^ ^49^ ^)^ Indeed, recent studies have confirmed the presence of endotoxin in NASH livers. Patients with NASH exhibited higher circulating endotoxin concentrations than patients with simple steatosis, similar to accumulation in hepatocytes, which was accompanied by increased numbers of toll‐like receptor 4–positive hepatic macrophages.^(^ ^50^ ^)^ Further human evidence for intrahepatic endotoxin is derived from another recent study reporting an increase in endotoxin in the portal tract.^(^ ^51^ ^)^ Experimental evidence also indicated that endotoxin‐producing strains such as *Enterobacter cloacae* B29, *Escherichia coli* PY102, and *Klebsiella pneumoniae* A7 promoted NAFLD in germ‐free mice on a high‐fat diet.^(^ ^52^ ^)^ Moreover, ethanol‐producing *Klebsiella pneumoniae* strains isolated from patients with NAFLD caused fatty liver disease in mice after oral gavaging.^(^ ^53^ ^)^ Due to space constraints, we do not discuss the important role of an impaired intestinal barrier in the pathogenesis of obesity‐related disorders and NAFLD.^(^ ^54^ ^)^ Vice versa, numerous experimental and clinical studies are targeting the intestinal microbiota to modulate susceptibility to NAFLD, as excellently reviewed recently^(^ ^55^ ^)^ (see Fig. 1). In conclusion, overwhelming evidence from the last decade underpins a major role of the intestinal microbiota in the pathogenesis of NAFLD.

#### Tissue and Systemic Microbiome: Paving an Avenue for the Gut–Liver or AT–Liver Axis?

A very exciting and rapidly evolving topic is the concept of a tissue (i.e., liver, AT) and circulating (i.e., blood) microbiome that expands beyond intestinal dysbiosis in NAFLD. Bacterial 16S ribosomal DNA can indeed be detected in blood, which correlated with risk of diabetes,^(^ ^56^ ^)^ similar to the report of a blood microbiome signature in NAFLD.^(^ ^57^ ^)^ In liver tissue, genetic material from bacterial taxa has been detected in two cohorts of patients with NAFLD.^(^ ^51^ ^)^ Sookoian and colleagues^(^ ^51^ ^)^ observed an increased abundance of genetic material from Proteobacteria in the liver of severely obese patients (similar to alterations in the gut microbiome). Indeed, a tissue‐specific microbiome signature can be observed in many different diseases including solid cancers,^(^ ^58^ ^)^ T2D, and obesity.^(^ ^59^ ^)^ Bacterial DNA reminiscent of the gut microbiome is also present in human omental, SAT, and VAT in obesity and T2D subjects.^(^ ^60^ ^)^ Importantly, in this study bacterial DNA in AT correlated with AT inflammation and immune cell infiltration. Schierwagen and colleagues detected a circulating microbiome in central, hepatic, and portal venous blood and peripheral blood from patients with cirrhosis receiving a transjugular portosystemic shunt^(^ ^61^ ^)^; and in some patients bacteria could be cultivated from these sites. As such, genetic material and, in some cases, live bacteria can be detected in the circulation and diseased liver/AT. Importantly, these studies do not provide implications for health or disease. However, they describe a compelling window of opportunity for research in organs that were previously considered sterile.

#### Metabolomics and NAFLD

Metagenomic sequencing and bacterial metabolite screens (metabolomics) allow insight into the functional repertoire of complex bacterial communities. The topic of metabolomics in NAFLD has been excellently reviewed recently.^(^ ^12^ ^)^ Hoyles and colleagues studied the plasma and urine metabolome, the fecal metagenome (i.e., the genetic repertoire of bacteria), and the hepatic transcriptome (i.e., the transcriptional profile) in human fatty liver disease of morbidly obese women.^(^ ^62^ ^)^ In this study, a fecal metabolite mainly derived from bacteria (i.e., phenylacetate) correlated with hepatic steatosis. Fecal transfer from obese women with high‐grade steatosis into mice caused hepatic steatosis, as did feeding phenylacetate to mice.^(^ ^62^ ^)^ In search of metabolites in the portal vein of subjects with metabolic dysfunction, Koh and colleagues discovered imidazole propionate, a microbially produced histidine‐derived metabolite.^(^ ^63^ ^)^ Patients with T2D exhibited higher circulating concentrations of this metabolite, and importantly, imidazole propionate affected insulin signaling by activation of p38 MAPK and phosphorylation of p62, which finally resulted in activation of mechanistic target of rapamycin.^(^ ^63^ ^)^ Levels of N,N,N‐trimethyl‐5‐aminovaleric acid, a metabolite of gut bacteria, are increased in the serum of patients with NAFLD; and this metabolite deteriorated experimental hepatic steatosis.^(^ ^64^ ^)^ Other microbial metabolites such as 3‐(4‐hydroxphenyl)lactate discriminated subjects with NAFLD with and without fibrosis, although the pathways involved remain unknown.^(^ ^65^ ^)^ A combination of 10 serum metabolites showed powerful discriminatory effects for detecting advanced fibrosis even with greater diagnostic accuracy than the Fibrosis‐4 index.^(^ ^66^ ^)^ Collectively, bacterial metabolites have increasingly recognized functions in health and disease in and beyond the intestine, which also emerges in human NAFLD. Future studies may reveal fascinating insights into the pathogenesis of NAFLD, which bears potential for clinical (e.g., diagnostic or therapeutic) use. Besides the discussed pathways, other aspects also might be relevant in gut–liver interactions. Colonic proinflammatory macrophages are able to regulate hepatic and adipose insulin sensitivity under a high‐fat diet.^(^ ^67^ ^)^ By generating macrophage‐specific and intestinal epithelium–specific chemokine receptor 2 KO mice, the authors demonstrated decreased infiltration by colonic macrophages, decreased intestinal permeability, improved glucose tolerance, and even a decrease in AT inflammation, highlighting a gut–AT axis.^(^ ^67^ ^)^ Interestingly, products of commensal bacteria such as L‐lactate or acetate affect enterocyte lipid pathways with altered lipid storage and oxidation.^(^ ^68^ ^)^ This could have distal effects, influencing lipid‐driven disorders such as atherosclerosis or NAFLD.

### Dietary Factors as a Driving Force in NAFLD: Proinflammatory Diets and Interactions With Gut Microbiota

Many dietary components exert a crucial impact on the development of NAFLD.^(^ ^69^ ^)^ Dietary factors might directly impact hepatic lipid metabolism^(^ ^70^ ^)^ or act through functional alterations of the gut microbiome, collectively referred to as “dysbiosis.”^(^ ^71^ ^)^ Various dietary components have so far been identified that can damage the liver. A Western diet characterized by high fat consumption and high intake of alcohol, salt, refrained grains, fructose, and red and processed meat is associated with an increased risk for developing and progressing NAFLD.^(^ ^69^, ^72^ ^)^ In healthy volunteers a high‐fat Western diet causes endotoxemia and low‐grade systemic inflammation.^(^ ^73^ ^)^ Trans‐fatty acids are unsaturated fatty acids of vegetables that are enriched in snack foods, fried foods, and margarines. Intake of such trans‐fat is negatively associated with all‐cause mortality and coronary heart disease mortality.^(^ ^74^ ^)^ The association with NAFLD is less well studied. Trans‐fat consumption has been related in a single large study with altered liver function tests and fatty liver index.^(^ ^75^ ^)^ Preclinical data propose that trans‐fats promote cholesterogenesis,^(^ ^76^ ^)^ and trans‐fat‐induced NASH is improved if trans‐fatty acids are deleted from the hepatic lipid pool, although the exact mechanisms remain elusive.^(^ ^77^ ^)^ Fructose has been linked to NAFLD by various clinical studies with an increased rate of liver fibrosis.^(^ ^78^ ^)^ Fructose intake triggers *de novo* lipogenesis in the liver, and this process also involves microbiota‐derived acetate.^(^ ^79^ ^)^ A recent study in 10 healthy subjects, however, with excess isocaloric fructose consumption for 8 weeks showed no detrimental metabolic effects including the liver.^(^ ^80^ ^)^ This raises some doubts as to whether fructose indeed damages the human liver to promote NAFLD. Wheat amylase trypsin inhibitors, a common dietary wheat component, activates intestinal macrophages^(^ ^81^ ^)^ and has also been linked to experimental NAFLD as its consumption aggravates liver and AT inflammation.^(^ ^82^ ^)^ The gut microbiota converts nutrients such as choline or carnitine into trimethylamine, which is metabolized by liver flavin monooxygenases to trimethylamine *N*‐oxide (TMAO),^(^ ^83^ ^)^ while discontinuation of red meat consumption reduced TMAO levels within 4 weeks.^(^ ^84^ ^)^ Numerous studies have demonstrated an association of TMAO with cardiovascular diseases.^(^ ^85^ ^)^ The relationship between TMAO with NAFLD is less well studied. In a trial with 60 subjects with biopsy‐proven NAFLD, a greater severity of NAFLD was associated with higher TMAO but lower betaine and betaine/choline ratio.^(^ ^86^ ^)^ Administration of TMAO aggravates murine hepatic steatosis on a high‐fat diet, potentially involving bile acid metabolism and farnesoid X receptor antagonism.^(^ ^87^ ^)^ Therefore, it is increasingly recognized that various common food components have proinflammatory potential and thereby might initiate and contribute to chronic inflammatory processes in and outside the gastrointestinal tract.

In contrast, various anti‐inflammatory food components might oppose the above‐described effects or serve as a pool for anti‐inflammatory metabolites. For example, indole, a tryptophan derivate processed by the gut microbiota, improves diet‐induced fatty liver disease in mice; and low serum levels correlate with human NAFLD.^(^ ^88^ ^)^ Importantly, several dietary interventional studies using either a low‐sugar diet,^(^ ^89^ ^)^ a carbohydrate‐restricted isocaloric diet,^(^ ^90^ ^)^ or a Mediterranean diet^(^ ^91^ ^)^ have demonstrated a beneficial effect on hepatic steatosis, while the impact on NASH and/or fibrosis is poorly defined. As such, specific dietary intervention is a promising strategy to treat NAFLD and obesity‐related disorders in the future.^(^ ^69^ ^)^

## Conclusions and Outlook

In the last decade, AT and the gastrointestinal tract emerged as critical drivers of inflammation and fibrosis in NAFLD. Despite the fact that inflammation plays such a key role in NAFLD pathogenesis, large randomized controlled trials specifically targeting inflammatory pathways are still lacking. Altered liver function involving, for example, hepatic insulin resistance and lipotoxicity are considered another hallmark of NAFLD.^(^ ^92^ ^)^ Interactions between the liver, AT, and the gut are bidirectional, as established by functional experiments using transgenic mouse models, which is exemplified by a plethora of studies that define critical cues in liver diseases such as loss of intestinal permeability, insulin resistance, the pathophysiology behind liver fibrosis, and bile acid metabolism.^(^ ^33^, ^54^, ^93^ ^)^ Collectively, evidence from the last decade corroborated our 2010 reported multiple parallel hits hypothesis,^(^ ^9^ ^)^ forming a concept that is based on mechanistic insights gained in animal models and descriptive clinical trials. A better understanding of this concept will result in clinical translation and novel therapeutics for this endemic disease.

## Author Contributions

All authors made substantial contributions to discussion of content and wrote, reviewed and edited the article.
